# Modes of Fatty Acid Desaturation in Cyanobacteria: An Update

**DOI:** 10.3390/life5010554

**Published:** 2015-02-16

**Authors:** Dmitry A. Los, Kirill S. Mironov

**Affiliations:** Institute of Plant Physiology, Russian Academy of Sciences, Botanicheskaya Street, Moscow 127276, Russia; E-Mail: ksmironov@gmail.com

**Keywords:** cyanobacteria, fatty acids, fatty acid desaturases, desaturation, lipids, taxonomy

## Abstract

Fatty acid composition of individual species of cyanobacteria is conserved and it may be used as a phylogenetic marker. The previously proposed classification system was based solely on biochemical data. Today, new genomic data are available, which support a need to update a previously postulated FA-based classification of cyanobacteria. These changes are necessary in order to adjust and synchronize biochemical, physiological and genomic data, which may help to establish an adequate comprehensive taxonomic system for cyanobacteria in the future. Here, we propose an update to the classification system of cyanobacteria based on their fatty acid composition.

## 1. Introduction

Cyanobacteria (formerly—blue-green algae) are considered as one of the most ancient groups of living organisms on Earth [1]. Studies of fossil microorganisms in Precambrian rocks (3.5–0.5 billion years ago) indicated the temporal morphological changes in fossil cyanobacterial communities caused by the irreversible changes of physicochemical conditions on Earth [2,3]. Different species of modern cyanobacteria inhabit almost all environments—from soil to fresh and sea waters, as well as such extreme habitats as hot springs, soda and salt lakes, *etc.* The morphology of some species, especially, extremophilic ones, resemble that found in fossils. Such species are called the relict cyanobacteria [2,4]. A comparison of artificial systems consisting of modern prokaryotes, including extremophilic cyanobacteria, and Proterozoic forms of cyanobacterial communities suggested that the cyanobacteria are very conservative and have changed insignificantly morphologically and, probably, physiologically during the past, at least, 2 billion years [4]. These negligible changes also refer to the membrane system of cyanobacteria, which is mainly determined by the lipids and fatty acid (FA) species.

The membranes of cyanobacteria are represented by the cytoplasmic (plasma) membrane and thylakoid membranes. Both membranes contain four major glycerolipids: monogalactosyldiacylglycerol (MGDG), digalactosyldiacylglycerol (DGDG), sulfoquinovosyldiacylglycerol (SQDG) and phosphatidylglycerol (PG). The molecular motion of these glycerolipids is determined mainly by the extents of unsaturation of the fatty acids that are esterified to the glycerol backbones [5]. The extent of unsaturation is, in turn, determined by the activity of fatty acid desaturases, the enzymes that introduce double bonds into specific positions of fatty-acyl chains of lipids [6]. Changes in the unsaturation of FAs affect various functions of membrane-bound proteins, such as the photochemical and electron-transport reactions that occur in thylakoid and cytoplasmic membranes of cyanobacterial cells [7].

FA composition of lipids of cyanobacteria is determined by the chain length (number of carbon atoms) and number of double bonds in these chains. In cyanobacteria, the FA chain length usually varies from C_14_ to C_18_. The number of double bonds in these chains may vary from 0 to 4 providing fully saturated FAs (with no double bonds), monoenoic (with 1 double bond), dienoic (with 2 double bonds), trienoic and tetraenoic (with 3 and 4 double bonds, respectively) FAs. FA composition of individual species of cyanobacteria is so conserved, that it may be used as a phylogenetic marker [8,9,10].

The system of classification of cyanobacteria according to their FA composition was proposed by Kenyon [11,12] and improved by Murata and co-workers [13]. According to this Kenyon-Murata classification, all cyanobacterial strains are divided into four distinct groups. Organisms in Group 1 introduce only one double bond at the ∆^9^ position of fatty acids (usually C_16_ or C_18_ FAs) esterified at the *sn-l* position of the glycerol moiety. In cyanobacteria of Group 2, the C_18_ stearic acid is desaturated at the ∆^9^, ∆^12^, and ∆^15^ [13] positions and the C_16_ palmitic acid is desaturated at the ∆^9^ and ∆^12^ positions. In Group 3, the C_18_ acid is desaturated at the ∆^6^, ∆^9^, and ∆^12^ positions. Finally, in Group 4, the C_18_ stearic acid is desaturated at the ∆^6^, ∆^9^, ∆^12^, and ∆^15^ positions (Table 1) [13].

The available experimental data on desaturation in cyanobacterial cells suggest that the ∆9-desaturase counts the carbon number from the carboxyl terminus, whereas the so-called ∆15-desaturase is, in fact, the ω3-desaturase, which counts the carbon number from the methyl-terminus [14]. Although significant progress has been made in understanding the molecular basis of regiospecific desaturation by soluble acyl–acyl-carrier-protein desaturases [15] the counting order of the acyl-lipid membrane-bound ∆12-desaturase is still under question. It is also important to note that the ∆15(ω3)-desaturase of the cyanobacterium *Synechocystis* sp. PCC 6803 cannot introduce double bonds into ∆^9^ monoenoic FAs, and it requires ∆^9,12^ dienoic substrate for its activity [16]. Here, we will use the ∆^x^ abbreviation system to simplify the designations.

Current conclusions on modes of FA desaturation in cyanobacteria are solely based on biochemical analysis of FAs and lipid classes [8,9,10,11,12,13,14]. Modern advances in sequencing techniques allowed determination of the whole genomes of various cyanobacterial strains. The genes for the specific acyl-lipid fatty acid desaturases have been identified in many cyanobacterial species [17]. This supports a need to update the previously postulated FA-based classification of cyanobacteria. Here, we propose an updated grouping of cyanobacteria, according to their FA composition, based on recent findings in cyanobacterial genomics and biochemistry.

**Table 1:** Fatty-acid composition of the total lipids from various cyanobacterial strains (adapted from Murata *et al.* 1992 [13]).

Organism		Fatty Acids										
		14:0	14:1	16:0	16:1	16:2	18:0	18:1	18:2	α18:3	γ18:3	18:4
			Δ^9^		Δ^9^	Δ^9,12^		Δ^9^	Δ^9,12^	Δ^9,12,15^	Δ^6,9,12^	Δ^6,9,12,15^
**Group 1**												
*Mastigocladus laminosus*	F	+	−	+	+	−	+	+	−	−	−	−
*Synechococcus* PCC 7942	U	+	−	+	+	−	+	+	−	−	−	−
*Synechococcus* PCC 6301	U	+	−	+	+	−	+	+	−	−	−	−
*Synechococcus lividus*	U	−	−	+	+	−	+	+	−	−	−	−
**Group 2**												
*Plectonema boryanum*	F	+	−	+	+	−	+	+	+	+	−	−
*Nostoc muscorum*	F	+	−	+	+	−	+	+	+	+	−	−
*Anabaena variabilis*	F	−	−	+	+	+	+	+	+	+	−	−
*Synechococcus* PCC 7002	U	+	−	+	+	−	+	+	+	+	−	−
**Group 3**												
*Arthrospira platensis*	F	+	+	+	+	−	+	+	+	−	+	−
*Synechocystis* PCC 6714	U	+	+	+	+	−	+	+	+	−	+	−
**Group 4**												
*Tolypothrix tenius*	F	−	−	+	+	−	+	+	+	+	+	+
*Synechocystis* PCC 6803	U	−	−	+	+	−	+	+	+	+	+	+

PCC—Number in Pasteur Culture Collection. F—filamentous species; U—unicellulae species.

## 2. Results and Discussion

### 2.1. Cyanobacteria of Group 1

The organisms of Group 1 synthesize only monoenoic FAs usually desaturated at Δ^9^ position. This group is presented by mesophilic and thermophilic strains of unicellular freshwater *Synechococcus* and *Cyanobacterium*, as well as by ramified filamentous heterocystous thermophilic *Mastigocladus laminosus* [18,19]. Previously, it was suggested that the number of double bonds in FA chains correlates with complexity of cyanobacterial cells [10,11,12], and filamentous strains are not distributed in Group 1. However, it appeared that *Mastigocladus laminosus* also belongs to Group 1 [13,20]. Thus, organisms that synthesize monoenoic fatty acids (usually, 14:1Δ^9^, 16:1Δ^9^, and 18:1Δ^9^) may be represented by unicellular and filamentous species (Table 2).

Genomic sequencing and biochemical analysis revealed that desaturation at Δ^9^ position may be performed by different isozymes of Δ9-desaturase. Some of these isozymes may be specific to *sn*-1 or *sn*-2 positions of the glycerol moiety [21].

**Table 2:** An updated classification of cyanobacteria on the basis of their fatty acid composition.

Organism	Fatty Acids										
	14:0	14:1	16:0	16:1	16:2 ^e^	18:0	18:1	18:2	α18:3	γ18:3	18:4
		Δ^9^		Δ^9^	Δ^9,12^		Δ^9^	Δ^9,12^	Δ^9,12,15^	Δ^6,9,12^	Δ^6,9,12,15^
**Group 1**											
*Synechococcus elongatus* PCC 7942 ^a^	−	−	+	+	−	+	+	−	−	−	−
*Mastigocladus laminosus*	−	−	+	+	−	+	+	−	−	−	−
*Synechococcus lividus*	−	−	+	+	−	+	+	−	−	−	−
*Synechococcus vulcanus*	+	+	+	+	−	+	+	−	−	−	−
*Cyanobacterium stanieri* PCC 7202 ^a^	−	−	+	+	−	+	+	−	−	−	−
*Cyanobacterium* sp. B−1200 ^b^	+	+	+	+	−	+	+	−	−	−	−
*Synechococcus cedrorum*	−	−	+	+	−	+	+	−	−	−	−
**Group 2**											
*Prochlorococcus marinus* ^c^	−	−	+	+	+	+	+	+	−	−	−
*Synechococcus* sp. (marine) ^d^	−	−	+	+	−	+	+	+	−	−	−
*Prochlorothrix hollandica* ^e^	+	+	+	+	+	+	+	+	−	−	−
**Group 3α**											
*Leptolyngbya boryana*	−	−	+	+	−	+	+	+	+	−	−
*Nostoc* sp.	−	−	+	+	−	+	+	+	+	−	−
*Anabaena* sp. ^f^	−	−	+	+	−	+	+	+	+	−	−
*Synechococcus* sp. PCC 7002 ^a^	−	−	+	+	−	+	+	+	+		−
*Gloeobacter violaceus*	−	−	+	+	−	+	+	+	+	−	−
*Trichodesmium erythraeum*	−	−	+	+	−	+	+	+	+	−	−
**Group 3γ**											
*Arthrospira platensis* B-256 ^b^	+	−	+	+	−	+	+	+	−	+	−
*Synechocystis* sp. PCC 6714 ^a^	−	−	+	+	−	+	+	+	−	+	−
*Synechocystis* sp. B-274 ^b^	−	−	+	+	+	+	+	+	−	+	−
**Group 4**											
*Tolypothrix tenius*	+	+	+	+	+	+	+	+	+	+	+
*Synechocystis* sp. PCC 6803 ^a^	−	−	+	+	−	+	+	+	+	+	+
*Lyngbya* sp. PCC 8106 ^a^	−	−	+	+	−	+	+	+	+	+	+
*Nodularia spumigena*	−	−	+	+	−	+	+	+	+	+	+

^a^ Number in Pasteur Culture Collection (PCC); ^b^ Number in the Collection of Microalgae and Cyanobacteria of the Institute of Plant Physiology RAS (IPPAS); ^c^ *Prochlorococcus* strains NATL1A, MIT 9211, MIT 9301, MIT 9303, MIT 9312, MIT 9313, MIT 9515, AS9601, CCMP1375, CCMP1986, *etc*; ^d^ Marine species of *Synechococcus*: strains BL107, CC9311, CC9605, CC9902, RCC307, RS9917, WH5701, WH7805, WH8102, *etc.*; ^e^ *Prochlorothrix hollandica* was reported to have ∆9- and ∆4-desaturase activities [22]; ^f^ At least, 9 species of *Anabaena* were studied [23].

The presence of six genes for the Δ9-desaturases in the genome of *Gloeobacter violaceus* [24] suggests that some isozymes may be specific both to the *sn*-position and to the carbon chain length of FAs. Since *Nostoc* [21] and *Gloeobacter* [24] do not belong to Group 1, one may suggest that multiple isoforms of the Δ9-desaturase are not typical to cyanobacteria of Group 1. Indeed, the type strains of unicellular freshwater *Synechococcus*, *Synechococcus elongatus* PCC 7942 (NCBI Reference Sequence NC_007604) and *Synechococcus elongatus* PCC 6301 (NC_006576), each have only one gene for the Δ9-desaturase. The appearance of 18:1Δ^9^ and 16:1Δ^9^ at *sn*-1 and *sn*-2 in these two strains [13] suggests that their Δ9-desaturases are not specific to the chain length of FAs and to the *sn*-position. However, the genome of a thermophilic unicellular cyanobacterium, *Thermosynechococcus elongatus* (similarly to filamentous *Nostoc* [21], and unusual unicellular “*single-membrane*” organism, *Gloeobacter* [24]) also carries several copies (three) of a gene for the Δ9-desaturase.

**Figure 1:**
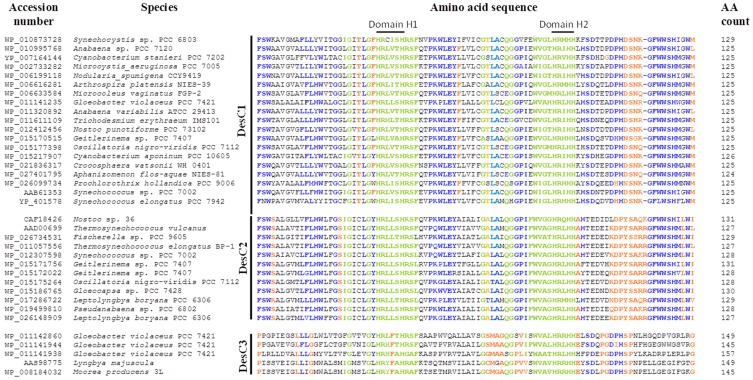

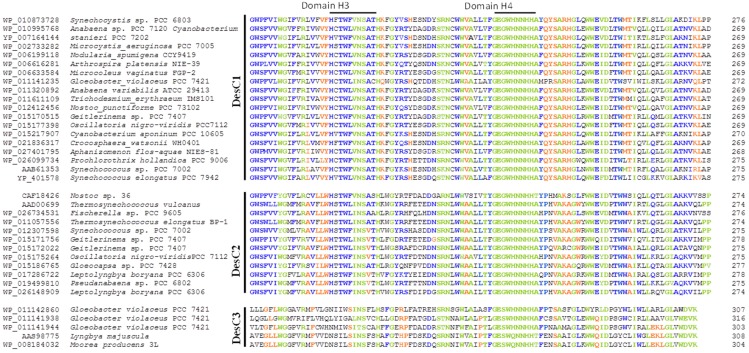
Alignment of partial amino acid sequences of the acyl-lipid fatty acid Δ9-desaturases from different cyanobacteria. The desaturases are clustered into three types of enzymes, DesC1, DesC2, and DesC3, according to their amino acid and functional features. Four conservative histidine-containing domains are marked. Amino acids identical or similar in all three groups of the Δ9-desaturases are shown in **green**; amino acids identical in two groups of desaturases are shown in **blue**; amino acids, which are unique for one of the desaturase groups, are shown in **orange**.

The alignment of amino acid sequences of Δ9-desaturases from various strains of cyanobacteria revealed that these enzymes can be classified into three groups (Figure 1). The first group, DesC1, is represented by the enzymes that are similar to the Δ9-desaturase, which is specific to *sn*-1 position of glycerolipids in *Synechocystis* sp. PCC 6803 and *Anabaena variabilis* [25]. Second group, DesC2, forms a cluster of enzymes homologous to the Δ9-desaturase, which is specific to *sn*-2 position in Antarctic *Nostoc* sp. 36 [21]. Differences in specificity of DesC1 and DesC2 to *sn*-position were demonstrated in accurate biochemical experiments [21,25]. The third distinct group of Δ9-desaturases, DesC3, is clustered by four amino acid sequences that were deduced from the genomic data of *Gloeobacter violaceus* [24] and two sequences of other cyanobacterial species.

At least four conservative His-containing domains found in these three groups of Δ9-desaturases. DesC1 and DesC2 were more similar to each other in amino acid sequences than DesC3 (Figure 1). First, second, and fourth His-containing domains (HRLXXHRSF, GHRXHH, GESWHNNHHA) are rather conservative in all three groups of Δ9-desaturases. The major differences in amino and sequences were observed in the domain 3 of DesC3 if compared to DesC1 and DesC2. The latter two have a very conservative third domain HFTWFVNSATH, while DesC3 has no His residues in this region. Conservative histidine residues function as coordinators of a diiron cluster in the active center of a desaturase that performs dehydrogenation reactions resulting in the formation of double bonds in the FA chains. Therefore, the positioning of His residues affects the specificity of FA desaturases in terms of a chain length and a position of desaturation [26]. The structural basis for positional specificity of desaturases is unknown. It might appear that the ability of desaturases to recognize a certain *sn*-position is similar to that of glycerolipid acyltransferases, in which a H(X)_4_D motif is a critical component for the enzyme’s activity [27].

The specificity of DesC1 and DesC2 to *sn*-1 and *sn*-2 positions have been documented [21,25], the specificity of DesC3 group of Δ9-desaturases was not studied experimentally. Therefore, the exact function of this type of enzymes is unknown. Chi *et al.* [17] found that this group of desaturases resembles a large family of membrane-associated Δ5- or Δ9-desaturases. Analysis of FA composition of *Gloeobacter violaceus* did not reveal any Δ5-desaturated FAs [28,29]. So, this should be some Δ9-desaturase with yet unraveled activity and specificity.

### 2.2. Cyanobacteria of Group 2

Previously, cyanobacteria that produce only mono- and dienoic FAs were unknown [13]. Therefore, Group 2 contained cyanobacteria capable of producing trienoic α-linolenic acid, 18:3Δ^9,12,15^ (*Anabaena*, *Nostoc*, *Gloeobacter violaceus*, *etc.*). Now we know a number of organisms that desaturate C_18_ and C_16_ FAs at positions ∆^9^ and ∆^12^ to produce mono- and dienoic fatty acids.

Genomes of these organisms contain genes for Δ9- and ∆12-desaturases. These are, mainly, representatives of marine species, *Prochlorococcus* and *Synechococcus*. We propose to allocate these cyanobacteria into Group 2 (Table 2). The analysis of lipids and FA composition of these organisms is still limited and requires detailed studies. In some plant, fungi, protist, and animal species, FA desaturases may possess bifunctional activities; one enzyme may catalyze two reactions, for example, the formation of double bonds at ∆^12^ and ω^3^ (∆^15^) positions [22,30]. Such bifunctional enzymes have not been yet reported in cyanobacteria. However, to confirm their absence, more experimental evidence is necessary on lipids and FAs for cyanobacteria of Group 2.

The freshwater filamentous *Prochlorothrix hollandica* differs from other cyanobacteria by the presence of light-harvesting chlorophyll *a*/*b* binding antenna and by the absence of phycobilins. *Prochlorothrix hollandica* is known as a C_14_-rich organism, which contains 5% of 14:0 and 30% of 14:1∆^9^ in lipids [31]. *Prochlorothrix*, together with ∆9-desaturase, has the unique ∆4-desaturase activity and produces unusual 16:1∆^4^ (25%) and 16:2∆^4,9^ (10%) FAs [31]. The genetic data for the cyanobacterial ∆4-desaturase is still unavailable. Nevertheless, the presence of high amounts of 16:2∆^4,9^ (and the complete absence of 18:2 FAs) should place *Prochlorothrix hollandica* to a special position in Group 2 of cyanobacteria, which are capable of synthesizing the dienoic FAs.

### 2.3. Cyanobacteria of Group 3

According to previously proposed classification, the cyanobacterial strains that synthesize trienoic α-linolenic acid, 18:3∆^9,12,15^ were assigned to Group 2. These organisms have three distinct FA desaturase activities: Δ9-, ∆12- and ∆15-desaturases. Organisms of a former Group 3 also have three distinct desaturases, but, instead of ∆15, they introduce a third double bond at position ∆^6^ and produce trienoic γ-linolenic acid, 18:3∆^6,9,12^, as a final product of desaturation.

We propose to combine all organisms that produce trienoic FAs as the final products of desaturation into Group 3, which will be divided into two subgroups—Group 3α and Group 3γ—according to the final product of desaturation—α- or γ-linolenic acids (Table 2).

Cyanobacteria that belong to a newly proposed Group 3α produce α-linolenic acid, 18:3∆^9,12,15^. These species (*Leptolyngbya boryana* (formerly, *Plectonema boryanum*), *Gloeobacter violaceus*, *Anabaena* sp., *Synechococcus* sp. PCC 7002, *Trichodesmium erythraeum*, some *Nostoc* species) are characterized both genetically and biochemically.

Genome sequencing of these species confirmed the presence of genes for the specific ∆9-, ∆12-, and ∆15-desaturases [24,32,33,34]. Lipid and FA analysis revealed the presence of 16:0, 16:1∆^9^, 18:0, 18:1∆^9^, 18:2∆^9,12^, and 18:3∆^9,12,15^ FAs [13,23,29,35,36,37,38].

The presence of a single strain of marine *Synechococcus* in this group (namely, *Synechococcus* sp. PCC 7002) raises a question about possible diversity of this genus in terms of FA composition. Table 2 clearly demonstrates that freshwater *Synechococcus* strains synthesize monoenoic FAs and belong to Group 1, whereas marine *Synechococcus* strains synthesize dienoic FAs and belong to Group 2. Alternatively, it may raise a question about the correct assignment of a strain PCC 7002 to a genus of *Synechococcus*.

Cyanobacteria of Group 3γ are capable of synthesizing the γ-linolenic acid, 18:3∆^6,9,12^. These organisms have three distinct FA desaturase activities: Δ6-, ∆9- and ∆12-desaturases. These cyanobacteria are represented by species of filamentous *Arthrospira* (*Spirulina*), unicellular *Synechocystis* sp. PCC 6714, and *Synechocystis* sp. IPPAS B-274.

The genomic and biochemical data for *Arthrospira* [29,39,40,41] and *Synechocystis* sp. PCC 6714 [13,42] are available, which support the positioning of these strains to Group 3. *Synechocystis* strains PCC 6714 and PCC 6803 are thought to be closely related species [42]. However, unlike *Synechocystis* PCC 6803 (Group 4, see below), *Synechocystis* sp. PCC 6714 lacks a gene for the ω3(Δ15)-desaturase [42], and it cannot synthesize α-linolenic and/or stearidonic acid.

### 2.4. Cyanobacteria of Group 4

Cyanobacteria of Group 4 have four acyl-lipid fatty acid desaturases and they can synthesize tetraenoic stearidonic acid, 18:4∆^6,9,12,15^, from C_18_ saturated stearic acid. In a model strain, freshwater unicellular *Synechocystis* sp. PCC 6803, synthesis of α-linolenic and stearidonic acids is temperature-dependent and occurs only at low temperatures (15–25 °C) [43]. Therefore, biochemical analysis cannot reveal 18:3α and 18:4 FAs in cells grown at optimal temperatures (30–36 °C). Genome sequencing [44] together with gene expression analysis [45] demonstrated the presence and expression of genes for Δ6-, ∆9-, ∆12-, and ∆15(ω3)-desaturases in *Synechocystis* sp. PCC 6803. And besides, the gene for ∆15(ω3)-desaturase was active only at low temperatures [45].

Similarly, genome sequence analysis of the marine filamentous cyanobacteria *Nodularia spumigena* and *Lyngbya* sp. PCC 8106 revealed the presence of genes for Δ6-, ∆9-, ∆12-, and ∆15(ω3)-desaturases [17]. Thus, these cyanobacteria would potentially produce α-linolenic, γ-linolenic, and stearidonic acids, the latter as a final product of desaturation.

Solid biochemical evidence is available for freshwater filamentous *Tolypothrix* species that confirms the presence of tri- and tetraenoic C_18_ FAs [12,13,29]. Recent lipid analysis of two strains, *Tolypothrix tenuis* and *Tolypothrix distorta* revealed previously undetected positional isomer of stearidonic acid, 18:4∆^3,6,9,12^ [46]. This so-called, γ-stearidonic acid was present in cells nearly in trace amounts. If this data is confirmed, it would be challenging to find a new cyanobacterial desaturase with ∆^3^ specificity. The complete genomic sequence of *Tolypothrix* may clarify whether a fifth, yet unknown, desaturase exists in cyanobacteria, or a double bond at position ∆^3^ is formed due to non-specific activity of ∆15(ω3)-desaturase on C_16_ FA, which is further elongated to C_18_.

### 2.5. Adaptive and Taxonomic Impact of Cyanobacterial Fatty Acid Composition

Cyanobacteria are characterized by rather limited set of FAs in their lipids: C_14_-C_18_ FAs with 1–4 double bonds. However, they have diverse phenotypes, and they inhabit very diverse environments, which, in many cases, are highly extreme. Fatty acid composition can be used to characterize different species of cyanobacteria, although the exact taxonomic meaning of FA composition is not completely understood. The organization or complexity of cyanobacterial cells (unicellular or filamentous) does not correlate with FA composition. A number of double bonds in FAs correlates instead with temperature of the environment. Thermophilic unicellular species usually have monoenoic FAs, whereas mesophilic or psychrophilic unicellular species produce polyunsaturated FAs, which help them to survive at low temperatures by adjusting the membrane fluidity [7]. Thermophilic filamentous species adjust the membrane fluidity by the inhibition of 16:0 acid elongation and by enhancement of the monoenoic 16:1 acid synthesis [29]. In mesophilic species, both mechanisms—accumulation of 16:1 and desaturation—may be active. In mesophilic filamentous *Anabaena variabilis*, a drop in temperature leads to accumulation of C_16_ in the dark, and to formation of polyunsaturated FAs (mainly, C_18_) in the light [29,47].

Fatty acid composition may be used to clarify the taxonomic position of a certain cyanobacterial strain. Thus, for example, it is rather surprising to find the representatives of genus *Synechococcus* (*Synechococcus* sp. PCC 7002) in diverse Groups 1, 2, and 3. Several authors noticed that the strains comprising the *Synechococcus* genus seem to be polyphyletic, and they suggested that this genus should be separated into different groups [48,49].

In general, the taxonomy of cyanobacteria is complicated and unclear [19,50]. The easiest and mostly used profiling technique employs the 16S rRNA gene sequence clustering. However, this simplified approach often leads to false assignments of strains and incorrect annotations. A more promising way to classify cyanobacterial strains is a polyphasic approach, which takes into consideration molecular, morphological, biochemical, and physiological characteristics of individual cultures and strains [51,52]. Recent developments in genome sequencing techniques provide a powerful tool for genetic profiling of cyanobacterial strains implying that sequence annotations are accurate. In such a polyphasic approach, the fatty acid composition is still a valuable marker to the cyanobacterial taxonomy.

## 3. Conclusions

The taxonomic system of cyanobacteria is developing according to combined multiple markers, including molecular, biochemical, ultrastructural, phenotypic and ecological data. The previously proposed system of biochemical classification of cyanobacteria according to their FA composition [11,12,13] is also changing. Here, we propose an update to this system according to newly available genomic and biochemical data. The basis of the system remains unchanged: cyanobacteria are grouped according the number of double bonds in their FAs. The major improvements are as follows. (1) The replacement of organisms in a previous “Group 2” with a new “Group 2” represented mainly by marine unicellular species, which are characterized by the presence of Δ9- and Δ12-desaturases and are capable of producing 16:2 or 18:2 FAs as the final product of FA desaturation. (2) Organisms previously assigned to Group 2 are transferred into Group 3, Subgroup 3α. Strains in this group are characterized by the presence of Δ9-, Δ12-, and Δ15(ω3)-desaturases, and they synthesize 18:3 α-linolenic acid as a final product of FA desaturation. (3) Organisms of the former “Group 3” (they have Δ6-, Δ9-, and Δ12-desaturases, and they synthesize 18:3 γ-linolenic acid) remain in Group 3, but placed into Subgroup 3γ. Group 1 (includes organisms that have Δ9-desaturase(s) and produce only monounsaturated FAs), and Group 4 (organisms with four FA desaturases, namely Δ6-, Δ9-, Δ12-, and Δ15(ω3)-desaturases, which may synthesize 18:4 stearidonic acid) remain unchanged.

These changes in FA-based classification system are necessary in order to adjust and synchronize biochemical, physiological and genomic data, which may help to establish an adequate comprehensive taxonomic system for cyanobacteria in the future.
